# Murine leukemia virus RNA dimerization is coupled to transcription and splicing processes

**DOI:** 10.1186/1742-4690-7-64

**Published:** 2010-08-05

**Authors:** Stéphan Maurel, Marylène Mougel

**Affiliations:** 1 Université Montpellier 1, Centre d'études d'agents Pathogènes et Biotechnologies pour la Santé (CPBS), CNRS, UMR 5236, 4 Bd Henri IV, 34965 Montpellier, France

## Abstract

Most of the cell biological aspects of retroviral genome dimerization remain unknown. Murine leukemia virus (MLV) constitutes a useful model to study when and where dimerization occurs within the cell. For instance, MLV produces a subgenomic RNA (called SD') that is co-packaged with the genomic RNA predominantly as FLSD' heterodimers. This SD' RNA is generated by splicing of the genomic RNA and also by direct transcription of a splice-associated retroelement of MLV (SDARE). We took advantage of these two SD' origins to study the effects of transcription and splicing events on RNA dimerization. Using genetic approaches coupled to capture of RNA heterodimer in virions, we determined heterodimerization frequencies in different cellular contexts. Several cell lines were stably established in which SD' RNA was produced by either splicing or transcription from SDARE. Moreover, SDARE was integrated into the host chromosome either concomitantly or sequentially with the genomic provirus. Our results showed that transcribed genomic and SD' RNAs preferentially formed heterodimers when their respective proviruses were integrated together. In contrast, heterodimerization was strongly affected when the two proviruses were integrated independently. Finally, dimerization was enhanced when the transcription sites were expected to be physically close. For the first time, we report that splicing and RNA dimerization appear to be coupled. Indeed, when the RNAs underwent splicing, the FLSD' dimerization reached a frequency similar to co-transcriptional heterodimerization. Altogether, our results indicate that randomness of heterodimerization increases when RNAs are co-expressed during either transcription or splicing. Our results strongly support the notion that dimerization occurs in the nucleus, at or near the transcription and splicing sites, at areas of high viral RNA concentration.

## Findings

The dimeric nature of the genome is strongly conserved among *Retroviridae*, underlying the importance of RNA dimerization for virus replication. Packaging of two genome copies increases the probability of recombination events by template switching upon the reverse transcription, thus promoting genetic diversity [1]. Dimerization may play an additional role in the sorting of the viral full-length RNA (FL RNA) between different fates, including splicing, translation, and packaging [2]. RNA structural switches induced by dimerization might be responsible for such RNA versatility [3-8]. Dimerization and packaging of MLV unspliced RNAs are well documented with identification of the RNA *cis*-element (Psi) and its interaction with the *trans*-acting Gag factor [6,9-18]. Dimerization appears to be a prerequisite for genomic RNA packaging [19] and could participate in the selection of the genome among a multitude of cellular and viral mRNAs. However, where and when RNA dimerization occurs in cell have long remained unresolved [19-21], and constitute the aims of the present study.

Presumably, dimerization occurs in the cell prior to RNA packaging as supported by recent microscopy studies at single-RNA-detection sensitivity [22,23]. Moreover, the co-localization of Gag and FL RNA in the nucleus suggests that Gag might bind the FL RNA inside the nucleus [24-26]. Such a connection between Gag nuclear trafficking and genome packaging provides an attractive model for how retroviruses first recruit their genomes. The consequence of the nuclear RNA life on RNA packaging and presumably on RNA dimerization is also supported by genetic approaches [27-30]. For instance, transcription of two MLV RNAs expressed from a single locus favored their co-packaging while transcription from distant loci did not. Here, we undertook the same genetic approaches coupled with virion RNA capture assays (RCA) to determine whether transcription and splicing steps could impact RNA dimerization efficiency. We took advantage of a unique characteristic of MLV to produce a splice-associated retroelement (SDARE) [31].

**Figure 1:**
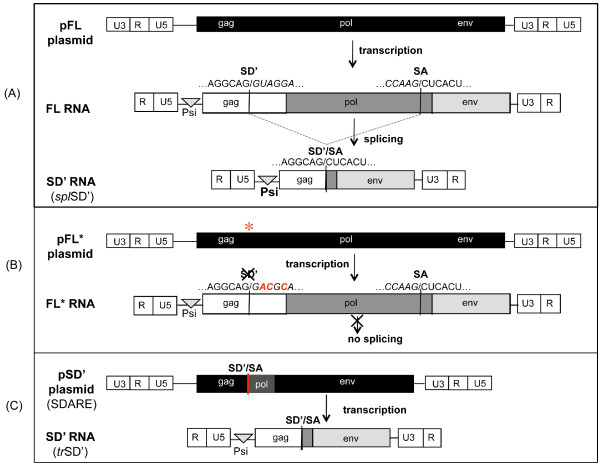
**Schematic representation of viral constructs and RNA expression**. The dimerization/packaging signal, Psi, is contained in all RNAs. (A) The pFL plasmid corresponds to Mo-MLV molecular clone (pBSKeco, a kind gift from FL.Cosset [59]) and generates FL RNA after transcription. The SD' RNA derives from splicing between an alternative splice donor site, designated SD', located within the *gag *gene, and the canonical splice acceptor site (SA). (B) The pFL* mutant contained three nucleotide substitutions in the SD' splice donor site that impaired the alternative splicing. (C) The pSD' plasmid allows prespliced SD' RNA production by direct transcription. After integration in the host genome, pSD' corresponds to SDARE.

In addition to the *env *mRNA, MLV produces an alternatively spliced 4.4-Kb RNA, called SD' RNA (Figure 1A). This alternative splicing recruits a splice donor site, SD', which is conserved among types C and D mammalian oncoretroviruses. Intact SD' is required for optimal virus replication and pathogenesis [32-35]. During the MLV life cycle, the SD' RNA shares all the characteristics of the FL RNA, since it goes through encapsidation, reverse transcription and integration steps. It acts as a defective retroelement (SDARE) that enables SD' RNA production via direct transcription by the cellular machinery, without the need for a splicing step [31]. Therefore, the SD' RNA can be generated via two different pathways, either by splicing of the FL RNA (*spl*SD') or by direct transcription of SDARE (*tr*SD').

The FL and SD' RNAs harbor the same Psi sequence responsible for their co-packaging. *In vitro*, the two RNAs harbored similar dimerization abilities and formed Psi-dependent heterodimers (FLSD') [36]. Analysis of virion content by RCA revealed that the SD' RNA was co-packaged with the FL RNA predominantly as heterodimeric forms [36]. This preferential dimerization of SD' RNA with FL RNA may influence recombination events since their association could restrict the interaction of FL RNA with other defective endogenous retroviruses or virus-like elements, and may have consequences in MLV pathogenesis [34,37,38]. Here, we took advantage of the propensity of the SD' RNA to form FLSD' heterodimers to study the impact of SD' transcription or splicing on MLV RNA dimerization.

### Transcription and dimerization

It has been reported that co-packaging of two MLV RNAs was dependent on the distance between their transcription sites [27,28]. These studies were based on the previous finding that stable co-transfection of two different plasmid DNAs lead to their integration as concatamers whereas a two-step stable transfection lead to two independent integration events [39-43]. These two transfection methods were validated for MLV-based vectors carrying different selectable markers. When two different viral RNAs were produced from tandem integrations by the one-step method, local and overlapping accumulation of both RNA transcripts were observed. In contrast, there was no co-localization of the RNAs generated by distinct transcription cassettes in the two-step approach [27,28].

**Figure 2:**
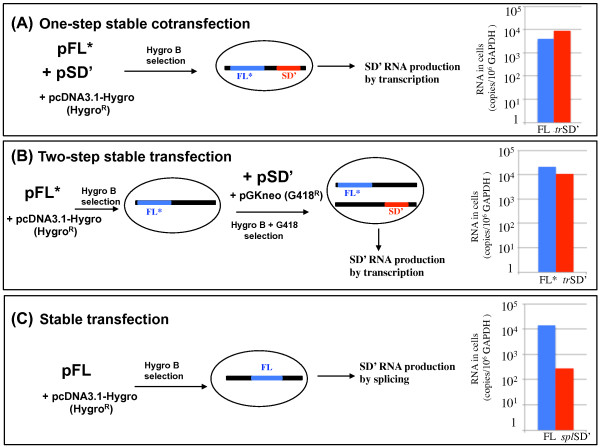
**Experimental strategy to study FLSD' heterodimerization in different cellular contexts**. Thick lines correspond to viral proviruses with genomic and SD' templates in blue and red, respectively. (A) One-step stable co-transfection of pFL* and pSD' allows concomitant integration of the two proviruses. Presumably, the transcription sites of the SD' and the FL RNAs are in close proximity on the chromosome. (B) Two-step stable transfections of pFL* and pSD' lead to sequential and independent integration events. SD' RNA is synthesized by transcription of a SDARE integrated in a site distant to that of FL provirus. (C) Stable transfection was performed with the replication-competent MLV molecular clone. SD' RNA is produced by splicing of the FL RNA. For each procedure, levels of the FL and SD' RNAs in stably transfected cells were determined by RT-QPCR. RNA copy numbers (cps) normalized to 10^6 ^cps GAPDH mRNA are given in the graphs.

**Figure 3:**
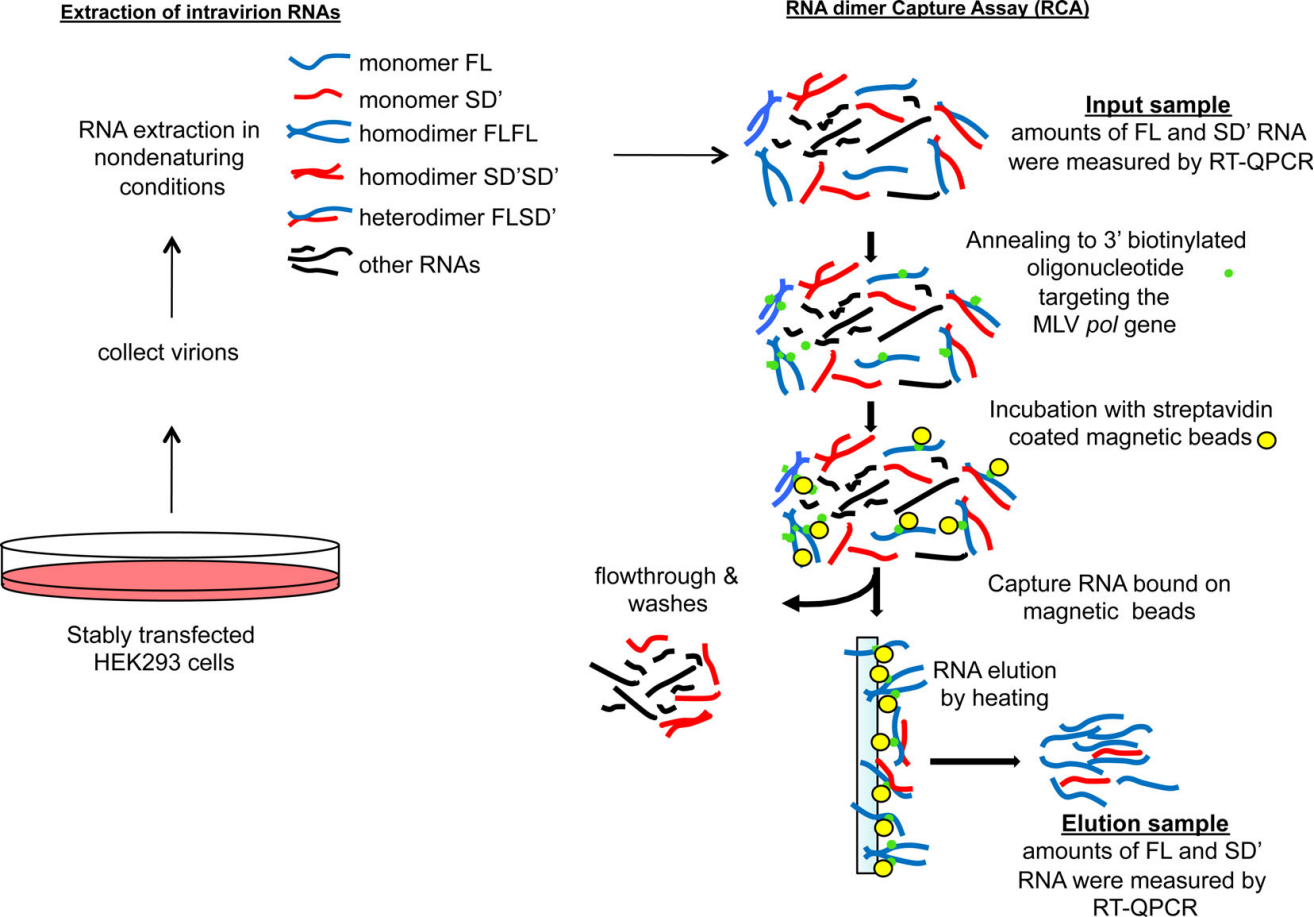
**Study of FLSD' heterodimerization by RNA Capture Assay (RCA)**. Details of the procedure were provided previously [36]. Briefly, two-days after transfection, RNAs were extracted from both cells and purified virions. An aliquot (1/5) of the RNA sample extracted from released virions was used for the input sample, whereas the rest (4/5) of the RNA sample was subject to the capture assay by using the 3'-biotinylated anti-MLV *pol *oligonucleotide (5' CAGTCTCTGTATGTGGGGCTTG 3'). Oligonucleotide-bound RNA was recovered by magnetic streptavidin-coated beads by using a magnetic stand. After several washes, the bound RNA was eluted by heating at 85°C for 5 minutes in water (elution sample). RNAs in elution sample were ethanol precipitated with 15 μg of carrier tRNA. Levels of FL and SD' RNAs were determined in cell extract, input and elution samples by specific RT-QPCR [36].

Here, we investigated whether the link between preferential co-packaging of two MLV RNAs and the proximity of their transcription sites was due to RNA dimerization [30]. To explore this possibility, we used the characteristic of MLV to produce two different proviruses, MLV and SDARE, which generate FL and SD' RNA transcripts, respectively [31]. To prevent the production of SD' RNA by splicing of the FL RNA, we used a mutant MLV carrying an inactive SD' site (pFL*) (Figure 1B). This mutation did not activate cryptic splicing sites and it slightly affected the MLV replication *in vitro *and *in vivo *(also called M1 or MSD1 in [32,34,35]). We used the same genetic approaches as previously validated, in which spatial positions of MLV proviral transcription sites are modulated by one versus two -step stable transfections [27,28,39-43]. Stably transfected 293-cell lines were established in which the FL and SD' (*tr*SD') RNAs were transcribed from pFL* and SDARE molecular clone (pSD'), respectively [31] (Figure 1C). The pFL* and pSD' plasmids were transfected together or sequentially to generate integrations in tandem or in distant loci, respectively (Figure 2AB). After selection, resistant colonies were pooled and RNA extracted from total cell extracts. Viral FL and SD' RNAs as well as the GAPDH mRNA were quantified by RT-QPCR as previously described [36]. The results indicate that the *tr*SD' and FL RNAs are equally transcribed in both contexts (Figure 2AB). The quantification of intracellular RNA dimers has long been an unresolved technical problem. Therefore, we measured the heterodimers in released virions, by using RNA Capture Assay (RCA), a tool designated to examine heterodimerization between two distinct RNAs [29]. All RCA steps were previously described for FLSD' heterodimerization analysis and were followed meticulously [36]. The major steps are briefly outlined in Figure 3. The FL RNA is used as a bait that was retained on the magnetic beads via a complementary biotinylated oligonucleotide. The SD' RNA was only captured via its association with FL RNA. Thus, SD' RNA presence in the elution can be used as a measure of heterodimerization. As described previously, the occurrence of heterodimerization was controlled by heat-denaturating the RNA samples before capture, in order to dissociate dimers. SD' RNA was no longer captured in the heat-treated samples [36]. The copy numbers of the FL and SD' RNAs were measured in the virion input and the elution fractions by specific RT-QPCR as previously described [31,36,44], and the SD' proportions in input and in elution samples are reported in Table 1. The elution/input ratios calculated for SD' reflect to some extent the heterodimerization efficiencies. Results from the two transfection procedures revealed that heterodimerization was ~30-times more efficient for proviruses integrated simultaneously, and presumably in tandem, than for proviruses integrated independently and likely in different loci.

**Table 1:** Comparative study of heterodimerization frequencies for SD' RNA produced in the different cellular contexts.

Experiment 1	SD' ORIGIN	**VIRION INPUT**^**(1)**^			**ELUTION**^**(2)**^			**%SD' **^**(4) **^**(elution/input) × 100**
			
		FL (cps)	SD' (cps)	%SD'	FL (cps)	SD' (cps)	**%SD' **^**(3)**^	
	
	transcription in same locus as FL	4.13E+06	4.24E+06	50.63	2.47E+05	4.83E+04	16.35	32.3
	
	transcription in distinct locus to FL	1.28E+08	3.60E+07	21.93	7.73E+06	1.60E+04	0.207	0.9
	
	splicing	9.80E+07	2.37E+05	0.24	6.34E+06	5.54E+03	0.087	36.1
								

**Experiment 2**	**SD' ORIGIN**	**VIRION INPUT**^**(1)**^			**ELUTION **^**(2)**^			**%SD' **^**(4) **^**(elution/input) × 100**
			
		**FL (cps)**	**SD' (cps)**	**%SD'**	**FL (cps)**	**SD' (cps)**	**%SD' ^(3)^**	
	
	transcription in same locus as FL	1.66E+07	1.47E+07	46.93	1.28E+06	2.54E+05	16.54	35.3
	
	transcription in distinct locus to FL	2.86E+08	3.44E+07	10.74	9.81E+06	1.86E+04	0.19	1.8
	
	splicing	8.57E+07	2.93E+05	0.34	6.21E+06	8.97E+03	0.144	42.4

Two independent RCA experiments were conducted from each HEK-293 cell line stably established as described in Fig.2. (1) Proportion of FL and SD' RNAs in virion input. The copies of FL and SD' RNAs determined in total virion samples before the RCA are indicated as well as the corresponding percent of SD' RNA in input. (2) The copies of captured FL and SD' RNAs quantified in total elution samples are indicated. (3) The % SD' in the elution was calculated as (SD'/(FL+SD')) × 100. (4) The FL RNA was the oligonucleotide-bound RNA, which should be retained by the beads and present in the elution. The SD' RNA was retained on the beads via its association with FL RNA and represents the heterodimer population. Based on the proportion of SD' in input, the proportion of SD' contributing to heterodimerization was calculated as the ratio of elution/input for SD' which corresponds to some extent to the heterodimerization efficiency.

To deduce the distribution of FLSD' heterodimers predicted for random RNA dimerization, we used the Hardy-Weinberg equation, as previously described for MLV RNA dimerization [29]. Predicted heterodimer proportions were compared to those determined experimentally (Table 2, column (3)). The two stably-transfected cell lines strongly differ in randomness of heterodimerization. For integrations in tandem, heterodimers formed at a frequency similar to that predicted from random RNA assortment. In contrast, for independent integrations, FL and SD' RNAs associated according to a non-random distribution, as previously reported [29,30].

**Table 2:** Comparison between the predicted and the measured heterodimerization efficiencies.

Experiment 1	SD' ORIGIN	Predicted distribution of homo- and hetero- dimers (1)			% of heterodimers captured in RCA (2)	randomness of heterodimerization
					
		FLFL (%)	SD'SD' (%)	FLSD' (%)	FLSD' (%)	prediction/experiment
	
	transcription in same locus as FL	24.4	25.6	50	32.7	1.53
	
	transcription in distinct locus to FL	60.9	4.8	34.2	0.41	83.4
	
	splicing	99.5	0.0006	0.5	0.17	2.9
						

**Experiment 2**	**SD' ORIGIN**	**Predicted distribution of homo- and hetero- dimers (1)**			**% of heterodimers captured in RCA (2)**	**randomness of heterodimerization**
					
		**FLFL (%)**	**SD'SD' (%)**	**FLSD' (%)**	**FLSD' (%)**	**prediction/experiment**
	
	transcription in same locus as FL	28.2	22	50	33.1	1.51
	
	transcription in distinct locus to FL	79.7	1.2	19.2	0.38	50.53
	
	splicing	99.3	0.001	0.7	0.29	2.41

(1) To deduce the distribution of FLSD' RNA heterodimers predicted for random RNA dimerization, we used the Hardy-Weinberg equation (A^2 ^+ 2AB + B^2 ^= 1), as previously described in details by Flynn et al. [29]. In this equation, A^2 ^and B^2 ^represent the percentage of FLFL and SD'SD' homodimers, respectively, and 2AB the FLSD' heterodimer population. Based on proportions of FL and SD' RNAs experimentally determined in virion input (Table 1), this equation allows the calculation of predicted percentages of AA (FLFL) and BB (SD'SD') homodimers in the viral population, and AB heterodimers (FLSD') represent the remaining percentage of the population. (2) The proportion of heterodimer experimentally determined by RCA was calculated from %SD' given in Table 1 as (2 × %SD'). (3) To determine the randomness of heterodimerization in the different HEK 293-derived cell-lines, the %FLSD' determined by the capture experiments were compared to that obtained by the prediction (predicted/measured).

These findings imply that MLV RNA dimer-partner selection occurs co-transcriptionally or within a pool of transcripts near the proviral templates. Our results correlate with previous studies showing the preferential co-packaging of MLV RNAs transcribed from the same chromosomal site [27,28]. Our finding indicates that RNA dimerization might be responsible for this preference.

### Splicing and dimerization

RNA splicing is spatially and functionally linked to transcription [45]. Therefore, the possibility of a correlation between splicing and dimerization, as already noted above for transcription and dimerization, was investigated. To test this new hypothesis, we determined the FLSD' heterodimerization efficiency with a SD' RNA issued exclusively from splicing (*spl*SD'). Cells were stably transfected with wild-type replication-competent MLV clone (here named pFL) and pcDNA-hygro plasmid (Figure 1A). After transcription, the FL RNA undergoes splicing to generate the SD' RNA. As expected, *spl*SD' RNA was less abundant than FL RNA in these cells (*spl*SD'/FL ratio is 1:50) (Figure 2C). Nevertheless, virion content analysis by RCA showed that spliced *spl*SD' RNA represented 0.1% of total elution leading to a heterodimerization efficiency of 36-42%. Interestingly, this efficiency was similar to that measured for co-expressed *tr*SD' and FL RNAs when their respective DNAs were cotransfected (Table. 1). Likewise, the *spl*SD' and FL RNAs segregated at a frequency close to that predicted from a random distribution (Table. 2).

Such a link between splicing and dimerization provides possible clues to the packaging process of spliced viral RNAs. Although the genomic RNA is preferentially packaged, the subgenomic RNAs are also specifically packaged into infectious HIV and MLV particles, although to a lower extent [31,46-48]. Such co-packaging of spliced and FL RNAs possibly involves heterodimerization. This model is supported by the ability of the MLV SD' spliced RNA to heterodimerize with the genomic RNA [36]. Note that HIV spliced RNAs were also able to dimerize *in vitro *[49,50]. It is still not clear how splicing contributes to dimerization. Dimerization might precede and somehow modulate splicing so that only one FL RNA molecule is spliced within FLFL homodimers, leading to asymmetrical dimers (FLSD'). Alternatively, the FL and SD' RNAs could associate during or soon after the splicing process is finished. This latter model correlates with our findings that splicing and co-transcription conferred similar heterodimerization efficiencies, implying the recruitment of a common mechanism for the two pathways.

Altogether our results showed that MLV RNAs preferentially dimerize when they undergo splicing or co-transcription. In contrast, the distance between transcription sites could hinder RNA dimerization. At least two non-exclusive hypotheses could explain these results. Host factors could play a role in dimerization [20,51]. For instance, transcription or splicing factors may confer a higher accessibility to the 5' end of the RNA including the dimer linkage structure (DLS) and thereby allows for better recognition of the DLS by the RNA partner and/or by Gag. Also, a direct role for an unidentified host candidate cannot be excluded. Similarly, nascent RNAs that are undergoing synthesis might adopt a more favorable conformation for dimerization compared to complete transcripts. In support of this model, dimerization occurred more efficiently for large synthetic MLV or HIV RNAs during *in vitro *transcription than post-synthesis [30,36,49]. Alternatively, co-transcription and splicing could enhance dimerization by providing high local RNA concentration in a subnuclear domain that facilitates RNA-RNA interactions. This mechanism is supported by previous studies showing that MLV RNA dimerization is dependent on RNA concentration *in vitro *[6,52]. Furthermore, it correlates with the nuclear accumulation of the viral FL RNA (75%) observed in MLV-producing cells [44].

Our results suggest that viral RNAs dimerize in the nucleus and presumably traffic out of the nucleus as dimers. Importantly, the MLV packaging signal (Psi) which overlaps the DLS, also contributes to nuclear export of the FL RNA [44,53]. Therefore, dimerization may impact on the RNA export pathway and determine the cytoplasmic fate of the RNA [54]. Dimers would be routed to virus assembly sites and packaged to serve as the viral genome, while monomers would be processed by the translation machinery to encode viral proteins. This would explain the occurrence of two functionally distinct pools of MLV FL RNA [55,56] and is supported by the nuclear localization of MLV Gag protein [24]. In agreement with this attractive model that we are testing in our laboratory, two articles were published upon completion of our manuscript, concluding that transient nuclear trafficking of Gag is required for RNA encapsidation in RSV or lentiviral particles [57,58].

## Authors' contributions

SM and MM conceived the study and analyzed the data. SM performed the laboratory work. MM wrote the manuscript. The authors read and approved the final manuscript.
